# Assessment of thiol/disulfide balance as an oxidative stress marker in children with β-thalassemia major

**DOI:** 10.12669/pjms.35.1.307

**Published:** 2019

**Authors:** Ahmet Guzelcicek, Gokhan Cakirca, Ozcan Erel, Abdullah Solmaz

**Affiliations:** 1 Ahmet Guzelcicek, Department of Pediatrics, Faculty of Medicine, Harran University, Sanliurfa, Turkey; 2 Gokhan Cakirca, Department of Biochemistry, Sanliurfa Mehmet Akif Inan Training and Research Hospital, Sanliurfa, Turkey; 3 Ozcan Erel, Department of Biochemistry, Faculty of Medicine, Yildirim Beyazit University, Ankara, Turkey; 4 Abdullah Solmaz, Department of Pediatrics, Faculty of Medicine, Harran University, Sanliurfa, Turkey

**Keywords:** β-thalassemia major, Disulfide, Ferritin, Oxidative stress, Thiol

## Abstract

**Objective:**

We aimed to investigate the oxidative stress status in children with β-thalassemia major (β-TM) by measuring native thiol (SH), disulfide (SS) and total thiol (SH + SS) plasma levels.

**Methods:**

This study was carried out from November 2017 to March 2018 at the Pediatric Hematology Clinic of the Harran University Medical Faculty Hospital. Blood specimens were collected from 100 participants, including 50 β-TM patients and 50 controls, and SH, SS and SH+SS levels were detected through a newly developed method.

**Results:**

SH, SS, SH+SS levels and SS/SH ratio were markedly higher in β-TM patients than in controls. In β-TM group, SH and SH+SS levels were positively correlated with age, albumin and total bilirubin. Serum ferritin level was positively correlated with SH, SH+SS, aspartate transaminase and alanine transaminase.

**Conclusions:**

We found that the SS/SH ratio was high in patients with β-TM, which shows increased oxidative stress. This ratio may be considered as a tool for the determination of oxidative status in such patients due to easily calculate, suitable for routine use and economical.

## INTRODUCTION

β-thalassemia major (β-TM) is a blood disorder that results in the deposition and precipitation of unmatched alpha globin chains in the erythrocytes due to impaired production of beta globin chains. This leads to the destruction of the erythrocyte membrane, ineffective erythropoiesis and, ultimately, severe anemia.^1^,^2^ Regular blood transfusions given to patients with β-TM, hemolysis and increased intestinal iron absorption lead to iron overload, and facilitate the production of reactive oxygen species (ROS).^3^,^4^ Oxidative stress, which results from a shift in the balance between ROS and the antioxidant defense system towards ROS, has been shown to play significant roles in the pathogenesis of diseases, including β-TM.^5^,^6^

Thiols, which include a sulfhydryl (-SH) group, constitute one of the defense systems against unfavorable effects of ROS, and are the main target of ROS, being oxidized by oxidant molecules to form reversible disulfide bridges. These formed disulfide bridges may be reduced to thiol groups. Oxidation-reduction reactions between the thiol and disulfide structures are crucial for the maintenance of the dynamic thiol/ disulfide balance (TDB) given the importance of this balance in several physiological processes in the organism, including apoptosis, detoxification of xenobiotics and antioxidant defense mechanisms, as well as in the stabilization of protein structures.^7^-^9^

A new colorimetric method to measure TDB tests in the blood became available in 2014. With this new method, TDB tests including native thiol (SH), disulfide (SS) and total thiol (SH + SS) levels can be quantified simultaneously. The other advantages of this method are that it is inexpensive, practical, simple, rapid, and suitable for routine use.^10^ Recent studies have reported that impaired TDB is associated with various disorders such as diabetes mellitus^11^, myocardial infarction^12^, familial Mediterranean fever^13^, and cancer.^14^ However, to our knowledge, TDB as an index of oxidative stress in children with β-TM has not been investigated by using a newly developed method. Therefore, this study was conducted to evaluate the TDB status in children with β-TM.

## METHODS

This study was conducted from November 2017 to March 2018 at the Pediatric Hematology Clinic of the Harran University Medical Faculty Hospital, Sanliurfa, Turkey. It included a total of 50 patients, including 28 males and 22 females, aged between two and 17 years with β-TM. β-TM diagnoses were based on a complete blood count, peripheral blood smear investigations and hemoglobin analyses performed by high-performance liquid chromatography. The patients were receiving regular erythrocyte transfusions and insufficient iron-chelating agents (deferoxamine or deferiprone or deferasirox). Patients with thalassemia intermedia or minor, endocrine disorder, heart disease or infection were excluded from this study. The control group consisted of 50 healthy children who were referred to hospital for a regular check-up and had no known disease. Our study was approved by Harran University Ethics Committee and written informed consents were obtained from the parents of all subjects.

### Specimen collection and analysis

Blood specimens were collected from the healthy controls and β-TM patients immediately before blood transfusions. Tubes containing ethylenediaminetetraacetic acid were used for measurement of TDB parameters and complete blood count, and gel separator tubes were used for the clinical chemistry and ferritin measurements. The collected blood specimens were centrifuged at 3000 rpm for 10 minutes, and then plasma and serum were separated. Plasma was immediately stored at -80°C until the analysis of the TDB parameters. Plasma levels of TDB in the patient and control groups were simultaneously tested via a novel colorimetric method developed by Erel O et al.^10^ In this method, reducible disulfide bonds (SS) are first reduced to free thiol groups (SH) using NaBH_4_. Secondly, the remaining NaBH_4_, which was used as a reducing agent, is removed with formaldehyde. Thirdly, 5, 5’-dithiobis-(2-nitrobenzoic) acid was used to measure the amount of total thiol (SH + SS). Dynamic SS content was determined using the [(SH + SS)-(SH)] /2) formula and the SS/SH ratio was calculated.

Serum ferritin level was measured with electrochemiluminescence method in a Cobas e602 analyzer (Roche Diagnostics, Germany). Hemoglobin and hematocrit were measured using a Cell-Dyn Ruby hematology analyzer (Abbott Diagnostics, United States). Serum levels of albumin, aspartate transaminase (AST), alanine transaminase (ALT), creatinine, total bilirubin and direct bilirubin were measured with conventional methods in a Cobas c702 analyzer (Roche Diagnostics, Germany).

### Statistical Analysis

The garnered data were analyzed using SPSS 21 software (SPSS Inc., Chicago), and p-value lower than 0.05 was considered significant. Kolmogorov Smirnov test was used to analyze whether the data were normally distributed. Student t-test or Mann-Whitney U-test was applied to compare continuous variables. The results were reported as mean ± SD or median (min-max). Categorical variables were compared using a Chi-Square test, with the data presented as numbers (%). The correlation between TDB tests and routine laboratory parameters (ferritin, ALT, AST, albumin, and bilirubin levels) was determined by Pearson or Spearman correlation test.

## RESULTS

The demographic characteristics and laboratory data of the β-TM patients and healthy controls are shown in Table-I. The mean age and gender distributions were not significantly different between the patient group and control group (P >0.05). Hemoglobin and hematocrit levels were significantly lower and the ferritin, AST and ALT levels were significantly higher in β-TM patients than in controls (P <0.05). Albumin and creatinine levels were similar in two groups (P >0.05).

**Table-I:** Demographic characteristics and laboratory findings of β-TM patients and healthy controls.

	β-TM (n=50)	Controls (n=50)	*p-*value
Age, years	7.46±4.10	7.38±3.98	0.921
Gender (male), n (%)	28 (56)	28 (56)	1.000
Hemoglobin, g/dL	8.37±1.04	13.57±1.13	<0.001
Hematocrite, %	24.16±3.04	39.20±2.79	<0.001
Ferritin, ng/mL	2608.5 (407.1-5362)	31.9 (16.8-145.8)	<0.001
ALT, U/L	29.5 (5.5-137.5)	15.9 (10.2-31.8)	<0.001
AST, U/L	32.6 (19.4-121.9)	25.9 (12.2-43)	<0.001
Creatinine, mg/dL	0.33 (0.11-1.15)	0.36 (0.14-0.63)	0.058
Albumin, g/dL	4.69±0.31	4.62±0.12	0.135
Total bilirubin, mg/dL	1.39 (0.43-4.30)	-	-
Direct bilirubin, mg/dL	0.37 (0.16-0.65)	-	-

β-TM: β-thalassemia major, ALT: Alanine aminotransferase, AST: Aspartate aminotransferase

When the parameters of TDB of the two groups were compared, SH (518.5±42.8 µmol/L vs 468.5±49.6 µmol/L, p<0.001), SH+SS (593.6±43.5 µmol/L vs 513.7±54.3 µmol/L, p<0.001), SS (37.5±10.2 µmol/L vs 22.6±7.9 µmol/L, p<0.001) levels and SS/SH ratio (7.31±2.14 vs 4.86±1.73%, p<0.001) were found to be higher in β-TM patients than in controls (Table-II).

**Table-II:** Thiol/disulphide balance parameters of β-TM patients and healthy controls.

	β-TM (n=50)	Controls (n=50)	p-value
Native thiol (SH), μmol/L	518.5±42.8	468.5±49.6	<0.001
Disulfide(SS), μmol/L	37.5±10.2	22.6±7.9	<0.001
Total thiol (SH+SS), μmol/L	593.6±43.5	513.7±54.3	<0.001
Disulfide/Native thiol (SS/SH),%	7.31±2.14	4.86±1.73	<0.001

β-TM: β-thalassemia major

As illustrated in Table-III, SH and SH+SS levels were positively correlated with age (r=0.562, p<0.001 and r=0.520, p<0.001), albumin (r=0.619, p<0.001 and r=0.580, p<0.001) and total bilirubin (r=0.357, p=0.011 and r=0.399, p=0.004) in β-TM patients. Ferritin level was positively correlated with SH (r=0.296, p=0.037), SH+SS (r=0.324, p=0.022), AST (r=0.304, p=0.032) and ALT (r=0.491, p<0.001). Such correlations were not observed in healthy controls.

**Table-III:** Correlation between thiol/disulfide balance and other parameters in β-TM patients.

	Native thiol (SH)		Total thiol (SH+SS)		Disulfide (SS)		Disulfide/Native thiol (SS/SH)	

	r	p	r	p	r	p	r	p
Age	0.562	<0.001*	0.520	<0.001*	-0.074	0.610	-0.223	0.120
Ferritin	0.296	0.037*	0.324	0.022*	0.070	0.631	-0.027	0.854
Hemoglobin	0.133	0.357	0.053	0.713	-0.167	0.248	-0.183	0.202
ALT	0.067	0.645	0.179	0.214	0.200	0.163	0.155	0.283
AST	-0.090	0.535	0.050	0.730	0.258	0.070	0.258	0.071
Albumin	0.619	<0.001*	0.580	<0.001*	-0.066	0.650	-0.219	0.126
Total bilirubin	0.357	0.011*	0.399	0.004*	0.160	0.267	0.062	0.668
Direct bilirubin	0.156	0.280	0.171	0.236	0.073	0.615	0.034	0.815

β-TM: β-thalassemia major, ALT: Alanine aminotransferase, AST: Aspartate aminotransferase. P<0.05 statistical significance.

## DISCUSSION

Continuous blood transfusions and hemolysis result in iron overload in the body tissues of β-TM patients. Iron overload in the organism leads to an increased generation of ROS and oxidative stress in β-TM patients.^3^,^4^ The extreme production of ROS can lead to cellular toxicity and dysfunction of organs such as heart, liver, pancreas and brain.^15^

Antioxidants (such as superoxide dismutase (SOD), glutathione peroxidase (GPx), glutathione, vitamins E) protect cells against oxidative damage by directly eliminating or preventing ROS production.^16^ Numerous studies have been conducted to determine the oxidant and antioxidant status in β-TM patients. Ghone RA et al.^6^ found higher malondialdehyde levels, an indicator of lipid peroxidation, and lower vitamin E and total antioxidant capacity in β-TM patients than in controls. They suggested that increased oxidative stress and depleted antioxidant levels play a critical role in pathogenesis of β-TM. Conversely, Kassab-Chekir A et al.^17^ demonstrated that SOD and GPX activities in β-TM patients were significantly higher than in control group. The significantly high levels of SOD and GPx may occur as a result of compensatory mechanism after increased oxidant stress.

Thiol, as another important antioxidant, plays a crucial role in defending the organism against free radicals through various mechanisms such as components of thiol/disulfide redox buffer, free radical quenchers, substrates for redox reactions.^18^ Kalpravidh RW et al.^19^ measured the levels of glutathione (GSH), intracellular antioxidant thiol, and the GSH disulfide form (GSSG), as an indicator of oxidative stress in β-thalassemia/Hb E patients. They found that GSH/GSSG ratio was significantly decreased in patients compared to control group. However, GSH forms a small part of the total body thiol pool. Thus, GSH and its oxidized disulfide form (GSSG) may not reflect the entire thiol/disulfide status of the body.

In the literature, there are some studies investigating the level of native thiol which is one side of the TDB in β-TM patients.^20^,^21^ Therefore, these studies did not provide any data on the TDB status. In our study, both sides of the TDB were measured with the new method. Thus, the TDB status can be evaluated cumulatively.

In this study, SH+SS, SH, ferritin, ALT and AST levels in patients with β-TM were higher than in control group, and SH+SS and SH were positively correlated with ferritin and bilirubin (an indicator of the hemolysis) in β-TM patients. In addition, serum ferritin levels correlated positively with ALT and AST. The significantly high ferritin level suggests iron loading in patients. Iron overload due to continuous blood transfusion may potentially cause hepatic toxicity in β-TM patients.^3^,^17^ Researchers observed that increased ferritin levels are associated with the formation of ROS, disruption of antioxidant system, and increased oxidative stress.^5^,^17^,^21^ Therefore, we think that the thiol levels increase as a compensatory response to damage due to free radicals.

We also found that the SS levels and SS/SH ratios were significantly higher in β-TM patients than in controls. In other words, TDB in patients with β-TM shifted towards the disulfide formation, which indicates that thiols are oxidized and transformed into disulfide bonds under oxidative stress. Our findings were consistent with the results of the study by Kalpravidh RW et al.^19^, suggesting decreased GSH/GSSG ratio and increased GSSG accumulation in β-thalassemia/Hb E patients. We believe that, SS/SH ratio may be used as an indicator of oxidative stress in β-TM.

Many studies have shown that levels of proinflammatory cytokines such as TNF-α, IL-1, IL-6, and IL-8 are increased in patients with thalassemia compared to controls.^22^,^23^ Moreover, Wanachiwanawin W et al.^24^ reported that elevated TNF-alpha and IL-1 levels may contribute to complications of beta-thalassemia/HbE disease. Another study revealed that, elevated proinflammatory cytokines returned to normal levels in thalassemia patients after treatment with chelation therapy.^25^ In our study, patients did not use chelation therapy regularly due to social and economic reasons. Therefore, we speculate that inflammation and insufficient chelation therapy may contribute to disturbance of TDB in thalassemia patients.

### Limitation of the study

This study was performed in a single center with small sample size. Also, the relationship between TDB tests and other oxidative stress markers was not investigated.

## CONCLUSIONS

To our knowledge, this is the first study demonstrating the TDB in pediatric β-TM patients. This study indicates that TDB shifts to the left, oxidized side in β-TM patients. More studies are needed to elucidate the role of TDB in the pathogenesis of β-TM.
